# Altered Expression of Circulating MicroRNA in Plasma of Patients with Primary Osteoarthritis and *In Silico* Analysis of Their Pathways

**DOI:** 10.1371/journal.pone.0097690

**Published:** 2014-06-05

**Authors:** Verónica M. Borgonio Cuadra, Norma Celia González-Huerta, Sandra Romero-Córdoba, Alfredo Hidalgo-Miranda, Antonio Miranda-Duarte

**Affiliations:** 1 Department of Genetics, Instituto Nacional de Rehabilitación (INR), Mexico City, Mexico; 2 Laboratorio de Genómica del Cáncer, Instituto Nacional de Medicina Genómica (INMEGEN), Mexico City, Mexico; Roswell Park Cancer Institute, United States of America

## Abstract

**Objective:**

To analyze a set of circulating microRNA (miRNA) in plasma from patients with primary Osteoarthritis (OA) and describe the biological significance of altered miRNA in OA based on an *in silico* analysis of their target genes.

**Methods:**

miRNA expression was analyzed using TaqMan Low Density Arrays and independent assays. The search for potential messenger RNA (mRNA) targets of the differentially expressed miRNA was performed by means of the miRWalk and miRecords database; we conducted the biological relevance of the predicted miRNA targets by pathway analysis with the Reactome and DAVID databases.

**Results:**

We measured the expression of 380 miRNA in OA; 12 miRNA were overexpressed under the OA condition (*p* value, ≤0.05; fold change, >2). These results were validated by the detection of some selected miRNA by quantitative PCR (qPCR). *In silico* analysis showed that target messenger RNA (mRNA) were potentially regulated by these miRNA, including genes such as *SMAD1*, *IL-1B*, *COL3A*, *VEGFA*, and *FGFR1*, important in chondrocyte maintenance and differentiation. Some metabolic pathways affected by the miRNA: mRNA ratio are signaling Bone morphogenetic proteins (BMP), Platelet-derived growth factor (PDGF), and Nerve growth factor (NGF), these latter two involved in the process of pain.

**Conclusions:**

We identified 12 miRNA in the plasma of patients with primary OA. Specific miRNA that are altered in the disease could be released into plasma, either due to cartilage damage or to an inherent cellular mechanism. Several miRNA could regulate genes and pathways related with development of the disease; eight of these circulating miRNA are described, to our knowledge, for first time in OA.

## Introduction

Osteoarthritis (OA) remains the most prevalent forms of arthritis and is a leading cause of disability. OA is well recognized as a multifactorial, degenerative joint disease with progressive loss of articular cartilage, joint space narrowing, osteophyte formation, subchondral sclerosis, and synovial inflammation, resulting in clinical symptoms such as pain and joint stiffness [1]. OA can occur in any joint, but OA of the knee is the most common type; in fact, it is considered that 6% of adults can be affected and that it is one of the most common reasons for total joint replacement [2].

Under physiological conditions, articular cartilage chondrocytes are responsible for a subtle balance between the synthesis of Extracellular matrix (ECM) components and type-II collagen, mainly aggrecan, and its degradation by proteolytic enzymes such as the Matrix metalloproteinases (MMP) [3]–[5] and the metalloprotease domain with the Thrombospondin type 1 motif family (ADAMTS) [6]–[8]. In OA, there is an imbalance of this process, resulting in an increased degradation process in the synthesis, leading to progressive articular cartilage loss, the hallmark of this entity [9]. OA is classified as primary when no discernible cause is evident and as secondary when a triggering factor is apparent, such as previous major injury, obesity, and particular occupational and sport activities, among others [2]. Several studies have demonstrated that primary OA has a strong genetic component [10]; however, recent evidence has made it apparent that epigenetic changes, altered expression of regulatory RNA and its consequent gene expression modifications could also participate in the biology of OA.

Epigenetic refers to heritable changes in gene expression that do not involve coding sequence modifications. The mechanisms of epigenetic regulation include DNA methylation, histone modifications, and small noncoding RNA [11]. MicroRNA (miRNA) are small noncoding RNA of ∼20–25 nucleotides (nt) in length that are transcribed in the nucleus by RNA polymerase II or III into a long precursor denominated primary-miRNA (pri-miRNA). This pri-miRNA is processed by the microprocessor Drosha-DGCR8 complex, an RNase III-type enzyme to generate a precursor of ∼70–100 nt, known as pre-miRNA, which is translocated into the cytoplasm via exportin 5. Pre-miRNA is processed by the ribonuclease Dicer, generating a miRNA duplex of ∼22 nt. Finally, one of the strands is incorporated into the RNA-induced silencing complex (RISC), where it is guided to its target mRNA [12], [13]. miRNA are involved in post-transcriptional gene expression regulation, targeting 30% of the coding genes through complementary base pairing between the miRNA and the 3′-Untranslated region (UTR) of the messenger RNA (mRNA) target, resulting in translational suppression or direct degradation of the mRNA [14]. To date, >1,600 human miRNA have been identified [15]–[18], and their contribution to diverse developmental processes and pathologies is well recognized [19]–.

The importance of miRNA in chondrocytes, therefore in OA, was highlighted by a Dicer-null mouse model whose growth plates exhibited a reduction in proliferating chondrocytes and accelerated differentiation into a hypertrophic type, resulting in severe skeletal growth defects and premature death [22].

Later, diverse genomic approaches have evaluated the differential expression of miRNA in OA vs a normal condition. Iliopoulos et al. [23] tested the expression of 365 miRNA in articular cartilage tissues from patients with OA; 16 miRNA were altered, representing one of the first miRNA signatures that distinguish osteoarthritic from normal chondrocytes. Additional data were generated during the following years, highlighting the importance of miRNA in the establishment and development of OA. Jones et al. [24] determined a set of 17 miRNA that contribute to cartilage alteration by profiling 157 miRNA, and these authors suggested that the altered expression of these miRNA could be the consequence of hypomethylated states in the miRNA promoter regions. Subsequently, some specific miRNA have shown important roles in OA; for example, down-regulation of miR-140 inhibits Interleukin-1beta (*IL-1β*) by inducing *ADAMTS* expression, miR-27b regulates *MMP-13* expression in human chondrocytes and is down-regulated in patients with OA, and miR-146a is overexpressed in patients with low-grade OA, suggesting its involvement in OA pathogenesis [25]–[27]. Because miRNA can be found in several fluids, recent studies have analyzed their expression to understand the biological process occurring in different pathologies. Murata et al. [28] investigated whether, in plasma and synovial fluid, miRNA could be used as possible biomarkers for Rheumatoid arthritis (RA) and OA, finding that some miRNA are capable of differentiating between both entities effectively. Based on this background, this study presents an exploratory analysis in a search for a set of differentially expressed miRNA in the plasma of patients with primary OA of the knee, and describes the biological significance of miRNA altered in OA based on an *in silico* analysis of their target genes.

## Materials and Methods

### Patients

The protocol was approved by the Committee for Ethics and Investigation of the Mexico City-based National Institute of Rehabilitation (INR). Study subjects were recruited at the INR Joint Rehabilitation Clinic. Patients with OA of the knee were >40 years of age with a clinical diagnosis of OA of the knee and radiologic scores of 2 and 3, with a Body mass index (BMI, kg/m^2^) of <27, without history of serious knee injuries, and without any other articular diseases. Controls were subjects aged >40 years, without a clinical diagnosis of OA of the knee, with a radiologic score of <2, who had undergone treatment for injuries not related with OA (tendon ruptures, contusions, bone fractures, etc.). Radiologic evaluation of all participants was performed by a sole trained observer who was blinded to the patients’ data. Grading of OA was assessed employing a 5-point scale according to the Kellgren-Lawrence radiographic scoring method in anteroposterior weight-bearing and lateral radiographs of the knees [29]. All subjects were interviewed to obtain the patient’s complete clinical history including general information, occupational, and sports activities, previous knee injuries, and clinical manifestations of OA, among others.

### Plasma Sample Processing

After obtaining the patient’s written informed consent, 5 ml of peripheral blood was collected into tubes containing Ethylenediaminetetraacetic acid (EDTA). Tubes were centrifuged at 1,800×*g* for 10 min at room temperature, and the supernatant was transferred into Eppendorf tubes. Then, the supernatant was centrifuged at 15,000*×g* for 5 min to precipitate cell debris and the final supernatant was stored at −80°C.

### Total RNA Isolation and Complementary DNA Synthesis

Total plasma RNA was isolated from 600 µl of pooled plasma of 73 samples using a mini miRNeasy kit (Qiagen, Inc, Valencia, CA) following the manufacturer’s protocol, and was eluted in 30 µl of RNase-free water. Total RNA concentration was determined by spectrophotometry with the NanoDrop 2000 spectrophotometer (Thermo Fisher Scientific, Inc.) and the RNA samples were stored at –80°C until further processing. miRNA complementary DNA (cDNA) was synthesized with the TaqMan microRNA Reverse Transcription Kit (Applied Biosystems, Foster City, CA, USA) and Megaplex RT primers (Human Pool A, Applied Biosystems) following the manufacturer’s protocol. This system allows for parallel Retrotranscription (RT) of 380 mature human miRNA from each plasma pool. Briefly, 90 ng of total RNA from the pooled plasma were retrotranscribed in the GeneAmp PCR System 9700 (Applied Biosystems) under the following cycling conditions: 40 cycles of 16°C for 2 min, 42°C for 1 min, and 50°C for 1 sec, followed by a final step of 80°C for 5 min to inactivate the reverse transcriptase. Then, in order to generate sufficient miRNA cDNA template, the cDNA was pre-amplified using Megaplex PreAmp primer Human Pool A and PreAmp MasterMix (Applied Biosystems, Foster City, CA, USA), following the manufacturer’s instructions.

### miRNA Expression Profiles

After the pre-amplification step, the products were diluted in 5 µl of Tris-EDTA (TE) buffer to perform miRNA gene expression profiles with the TaqMan Low Density Array ver. 2.0 plate A (Applied Biosystems, Foster City, CA, USA), which is a 384-well PCR-based microfluidic card with embedded TaqMan primers and probes in each well, for the 380 different mature human miRNA and 4 nucleolar RNA controls. The real-time PCR reaction was carried out according to the manufacturer’s protocol in a 7900 HT Fast Real-Time PCR system machine (Applied Biosystems, Foster City, CA, USA).

### TaqMan miRNA Assay for Independent Validation of Individual miRNA

Ten specific miRNA were tested on 27 independent patients’ plasma samples and on 27 control samples in 2-step PCR as part of miRNA expression validation. First, 25 ng of total RNA was isolated from 400 µl of the plasma samples and retrotranscribed in duplicate using specific miRNA primer and TaqMan probes (Applied Biosystems, Foster City, CA, USA). Then, Retrotranscription (RT) was conducted in a downscaled RT reaction of 5 µl, according to the protocol described by Kroh et al. [30] RT was carried out in a GeneAmp PCR 9700 System at 16°C for 30 min, at 42°C for 30 min, and at 85°C for 5 min. Then, real-time quantitative PCR (qPCR) was performed after the pre-amplification procedure (as previously described) with forward and reverse specific primers for each human miRNA (miR-16, -29c, -93, -126,-146a,-184, -186, -195, -345, and -885-5p) and control MammU6. These microRNA were chosen based on the differential expression between OA and normal samples and because of their statistical significance.

### Statistical Analysis

Descriptive statistics were applied to determine differential individual characteristics between patients with OA and control-group subjects by the Mann-Whitney *U* test for continuous variables and the Chi-square (*Χ^2^*)or Fisher exact test (two-tailed) for categorical variables. The Stata ver. 10.0 statistical software package (StataCorp, College Station, TX, USA) was utilized for the calculations.

Relative expression levels of miRNA were calculated using the Comparative delta delta Ct ( ΔΔCt) method [31], [32]. The Ct value (fractional cycle number at which fluorescence reaches the threshold) was determined utilizing an automatic baseline and a threshold of 0.2 across all of the plates. MammU6, an embedded control in TaqMan Human MicroRNA Arrays, was employed as an endogenous control for normalization because it has the most stable expression across all of the samples. Standard Student *t* test was used to test the differential expression of miRNA. A miRNA with a *p* value<0.05, a false discovery rate of 5%, and a fold change of ≥2 were labeled as differentially expressed. Unsupervised clustering analysis, using Pearson correlation and average linkage, was employed to identify the different subgroups defined by miRNA expression profiles. All of the previous analyses were performed with R software (HTqPCR, gplots) in the bioconductor platform.

### Computational Prediction of Potential miRNA Targets

The search for potential mRNA targets of differentially expressed miRNA was carried out by means of the miRWalk database using the integrated results from miRanda, PITA, and TargetScan algorithms and miRecords (www.mirecords.biolead.org). A miRNA was considered a potential target if it appeared in at least 3 of the algorithms utilized. In order to determine the biological relevance of the predicted miRNA targets, we conducted an enrichment pathway analysis with the Reactome (www.reactome.org) and DAVID (http://david.abcc.ncifcrf.gov) databases and plotted the most probable biological pathways in a heatmap plot. Another approach was to filter the mRNA target list through the data from the literature, including mRNA that had been already validated as altered miRNA targets in the disease; by utilizing this approach, only genes already related to the OA were included in the analysis, reducing the number of predicted target genes listed based on biological criteria.

## Results

### Differential Expression of miRNA Between OA and Normal Plasma Samples

**Figure 1:**
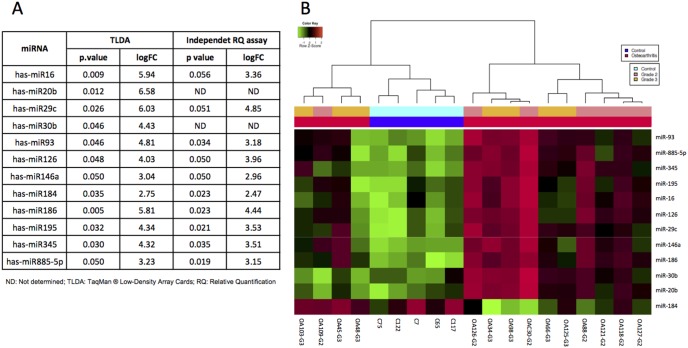
Profiling of plasma MicroRNA (miRNA) levels in patients with Osteoarthritis (OA). (A) Microarray analysis for miRNA levels was performed using RNA extracts from plasma of healthy subjects as control and patients with OA of different OA disease stages; (B) Unsupervised hierarchical clustering of the differentially expressed miRNA. Bright green: down-regulation; black, no change; bright red: up-regulation.

A Retrotranscription-PCR (RT-PCR) array analysis of miRNA in plasma was developed to identify a possible differential expression miRNA profile to characterize patients with OA with a radiologic grading score of 2 and 3 and healthy controls. Three hundred eighty miRNA were evaluated using TaqMan profiling Low Density Array (TLDA) in the plasma of 14 patients with primary OA of the knee and of 5 controls; their characteristics are depicted in Table 1. We observed the amplification of at least 65% of the total miRNA included in the array, in subjects with OA as well as in healthy subjects. When we performed the comparison of the study groups, we identified that 41 miRNA were found in both groups (Table S1). Of these, 26 showed differential expression, but only 12 were significantly deregulated (*p* value, ≤0.05, false discovery rate, 5%; fold change, >2), being overexpressed in the OA condition (Figure 1A and Table S1). Unsupervised hierarchical clustering analysis was conducted using the expression level of these 12 differentially expressed miRNA, resulting in clear differentiation of OA samples from normal samples in two different clusters. We did not observe differences between patients with OA of the knee with grades 2 and 3; likewise, statistically significant differences were found in miR-184 (*p* = 0.03); however, this shows clear heterogeneity along the samples (Figure 1B).

**Table:** **Table.1** Characteristics of the study sample.

	TLDA			Independent assay		
	OA (*n* = 14)	Controls (*n* = 5)	*P*	OA (*n* = 27)	Controls (*n* = 27)	*P*
Female (*n,* %)	10 (71.4)	5 (100.0)	0.1	24 (88.9)	22 (81.5)	0.4
Age [median (Min–Max), years]	55.7 (40.7–69.0)	47.5 (40.0–51.8)	0.1	55.6 (38.6–66.6)	52.9 (40.9–71.2)	0.5
BMI [median (Min–Max), kg/m^2^]	25.7 (19.0–28.1)	23.9 (21.1–24.9)	0.4	27.7 (19.9–35.9)	25.5 (18.3–33.5)	0.01
Kellgren-Lawrence grading						
0 (*n,* %)		5 (100.0)			27 (100.0)	
2 (*n,* %)	6 (42.8)			14 (51.8)		
3 (*n,* %)	8 (57.2)			13 (48.1)		

TLDA: TaqMan profiling Low Density Array; OA: Osteoarthritis; BMI: Body mass index.

### qRT-PCR Validation

To validate miRNA array results, quantitative Reverse transcription (qRT)-PCR singleplex was carried out to replicate the deregulated miRNA expression in 27 patients and 27 controls (Table 1). RT-PCR results confirmed overexpression of 10 miRNA, presenting a 3–4-Fold change (FC); miR-29c exhibited the highest FC and miR-184, the lowest, as observed previously in the TLDA assay (Figure 1A).

### Putative Target mRNA Genes of Differentially Expressed Circulating Plasma miRNA in OA

**Figure 2:**
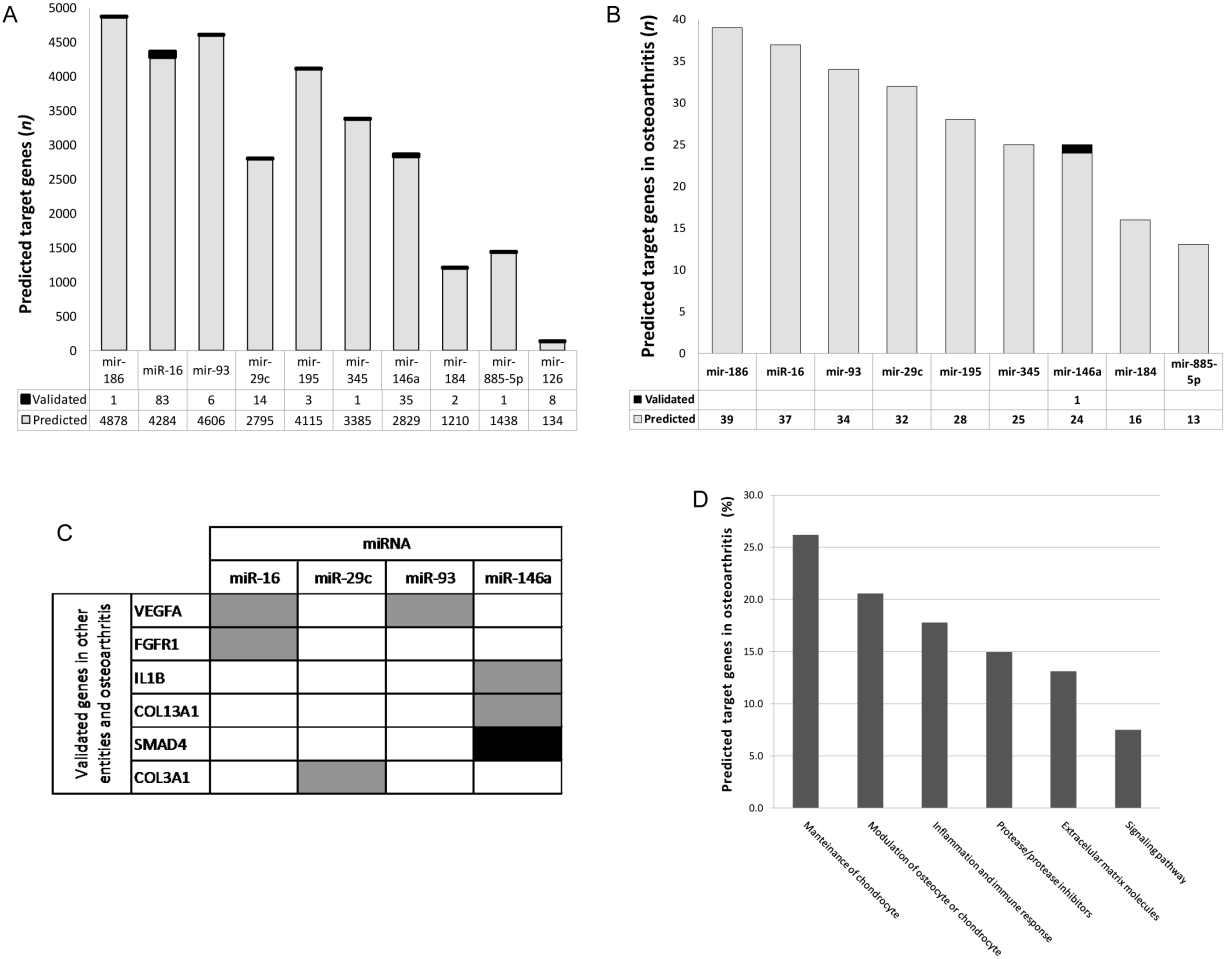
Transcripts targeted of microRNAs up-regulated in samples Osteoarthritis (OA). (A) Tabulation of the number of transcripts targeted of 10 miRNA; (B) Tabulation of the number of predicted targets for 150 genes involved in OA; (C) Predicted targets that have been confirmed previously in other entities and OA; (D) Percentage of predicted target genes involved in OA.

**Figure 3:**
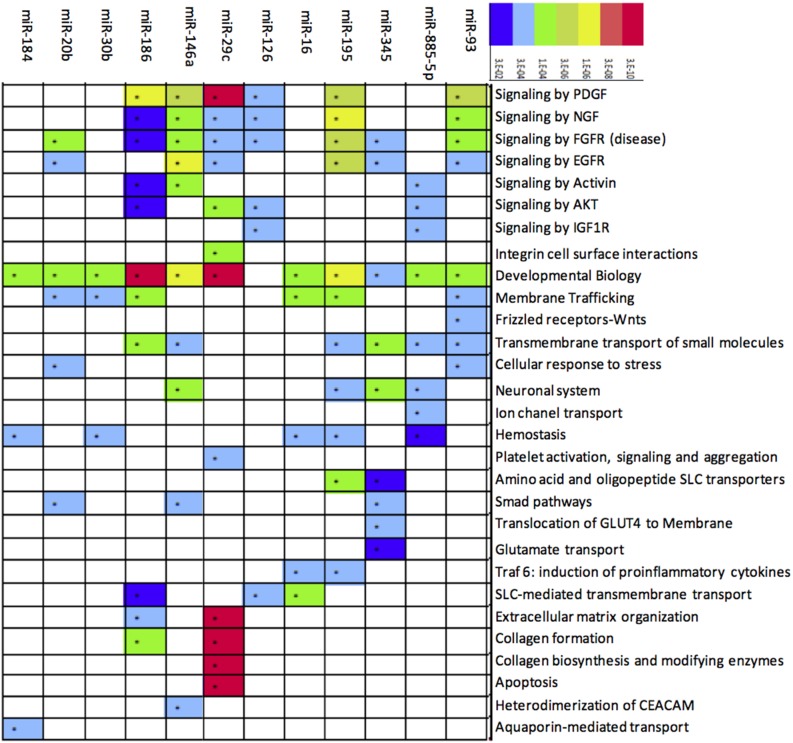
Pathways associated with microRNA (miRNA) up-regulated in Osteoarthritis (OA). Enriched biological processes among the 12 up-regulated miRNA. Red shows the e-value as more significant, while less significance is denoted in blue.

To determine potential miRNA targets, we carried out an *in silico* prediction analysis from mRNA human genome through the miRecords database. An mRNA was considered a potential target if it appeared in at least 3 of the algorithms utilized. Employing that criterion, 29,674 mRNA targets were predicted, of which 155 had already been validated by means of functional assays (Figure 2A). Those mRNA were filtered with the purpose of knowing those related with OA, which resulted in 102 possible mRNA targets for 9 of the 10 miRNA analyzed. Additionally, several of those miRNA had common targets (Figure 2B and Figure S1). Only miR-126 did not show any affinity for a possible target gene in OA. When we compared the group of miRNA putative targets in OA with the experimentally validated miRNA target miRecords database, we observed that at least 6 target genes are related with OA and have been validated in other entities. Interestingly, those 6 genes are regulated by 4 of the miRNA that we found in plasma and that are overexpressed in OA (miR-16, miR-29c, miR-93, and miR 146a). Only *SMAD4* has been validated exclusively in OA (Figure 2C). Target mRNA that showed affinity for miRNA encode molecules that belong to different pathways, such as those of chondrocyte maintenance, osteocyte modulation, or of chondrocyte inflammation and immune response, proteases and their inhibitors, Extracellular matrix (ECM) molecules, and signaling pathways (Figure 2D). Interestingly, some specific target genes were the following: Fibroblast growth factor receptor 1 (*FGFR1*); Histone deacetylase 4 (*HDAC4*); Fibroblast growth factor 2 (*FGF2*); Vascular endothelial growth factor A (*VEGFA*); Insulin-like growth factor I receptor (*IGF1R*); A Disintegrin-like and Metalloproteinase with Thrombospondin type 1 motif-5 (*ADAMTS5*); Tissue inhibitor of metalloproteinase 2 (*TIMP2*), and WNT1-inducible signaling pathway protein 1 (*WISP1*), all of which actively participate in OA development (Figure S1). Additionally, to define miRNA cellular impact, a pathway analysis was carried out through the Reactome and DAVID databases. Analysis showed miRNA involvement in diverse pathways; specifically within an OA context, it is noteworthy that some of these are involved in the establishment and development of OA, such as Platelet-derived growth factor (*PDGF*) and *FGFR*, ECM organization, collagen formation, and cellular biosynthesis and apoptosis (Figure 3).

## Discussion

Alterations of miRNA plasma profiles in response to various diseases have been previously reported [33]–[35], although the function of these extracellular circulating miRNA is not well understood. However, free cell miRNA may be a good biomarker candidate for diagnosis, prognosis, and therapy in different diseases. In the present study, our interest was to assess miRNA expression profiles in the plasma of patients with OA of the knee in comparison with those of healthy subjects.

Different from those described by other authors [23], [28], [36], we evaluated plasma samples of OA of grades 2 and 3 (early and intermediate OA); these stages are important in OA because they represent the beginning and progress of the entity. Interestingly, our study profiles noted the expression of 380 human plasma miRNA, identifying a set of 12 miRNA overexpressed. When we validated the expression levels of 10 miRNA by qPCR, we confirmed the high expression of 10 circulating miRNA in patients with OA. For these miRNA, at least eight miRNA are described, to our knowledge, for first time in OA as follows: *miR-29c, -93, -126, -184, -186, -195, -345,* and *-885-5p*. We also describe miR-16 and miR-146a in plasma that had been described previously in chondrocyte by other authors [27], [28], [37]. The circulating miRNA that we describe here could derive from several sources as a result of tissue destruction due to direct cartilage lesion, chronic inflammation, and apoptosis, or from tissue cells affected in the OA process that could release these miRNA. This could indicate that miRNA could be released into the extra-articular space of the chondrocyte, and it is possible that the type of miRNA depends on the disease stage, in a similar way to that reported by Montecalvo et al. [38], who showed that Dendritic cells (DC) communicate with their neighbor cells to amplify their function through diverse mechanisms. One of these mechanisms is transferring nanovescicles, denominated exosomes, which are loaded with different sets of microRNA according to the DC maturation stage. These authors also showed that exosomes can fuse with specific target cells, releasing their membranous and cytosolic contents into the DC cytosol [38]. For example, Murata et al. [28] analyzed the concentration of miR-16, miR-132, miR-146a, miR-155, and miR-223 in the plasma and in the Synovial fluid (SF) of patients with OA and RA. As in our study, the authors found deregulation of miR-16 and miR-146. These miRNA were detected in both plasma and SF samples; however, in plasma, the levels were higher than in SF [28]. In addition, Iliopoulos et al. [23] previously reported increased expression of miR-16 in chondrocytes of OA grades 3 and 4.

Another possibility is that the miRNA that we describe as deregulated in OA probably do not reflect the disease stage, possibly due to the origin of the miRNA, in that their intra- and extracellular profiles can differ substantially. In fact, we only found coincidence in two miRNA (miR-16 and miR-146a) among the profiles of the circulating miRNA that are described with the intracellular miRNA detailed in OA. This is interesting, because miRNA described as intracellular can also be found in extracellular form, which could be analyzed as possible biomarkers in later studies. On the other hand, their study in patients with OA is interesting due to the intra- and extracellular correlation that we are observing. One example of this is miR-146a. This small RNA is particularly interesting because its deregulation in OA has been consistently reported in several studies; for example, in Rheumatoid arthritis (RA), which is characterized by chronic inflammation, miR-146 was found overexpressed in synovial tissue after stimulation with the inflammatory cytokine Tumor necrosis factor alpha (TNF-α) and Interleukin-1β (IL-1β) [37]. In a study whose objective was to identify a molecular target of miR-146 in order to elucidate its function in chondrocytes during OA pathogenesis in an experimentally induced OA model, chondrocytes treated with IL-1β resulted in overexpression of miR-146a, accompanied by up-regulation of Vascular endothelial growth factors (*VEGF*) and inhibition of Smad4. These are interesting findings because they show that miR-146a may contribute to OA pathogenesis by increasing VEGF levels, which may in turn affect angiogenesis and inflammation, and because of the down-regulation of *Smad4* that is a common mediator of TGF-β, which has been shown to exert a protective effect on OA [39].

The remaining miRNA described could be regulating several mRNA that are directly related with OA. For example, in other entities or cellular models, it has been demonstrated that miR-16 and miR-93 targeted *VEGFA* and *FGFR1* [40]; thus, these miRNA could play a role in anomalous vascularization of articular tissue because they are regulating the expression of these growth factors [41]. In injured rat spinal cord, there is an increased level of miR-16, changing the expression of apoptotic target genes [42], and apoptosis is related with OA [43]. In renal fibrosis, up-regulation of miR-29c reduces the expression of collagens I, III, and IV [44]. In OA, there are no studies of miR-29c; however, *in silico* analyses suggest that *COL3A* could be its target and could be modulating ECM proteins, leading to cartilage damage.

The target genes of miRNA described in this paper could be regulators in several signaling pathways, including the following: Platelet-derived growth factor (*PDGF*), which plays a role in embryonic development, cell proliferation, cell migration, and angiogenesis, and Epidermal growth factor (*EGF*), which plays an important role in the regulation of cell growth, proliferation, and differentiation by binding to the EGF receptor (EGFR), a key role in multiple cellular processes such as apoptosis, cell proliferation, transcription, and cell migration. Protein kinase B, also known as *AKT*, plays a key role in multiple cellular processes such as apoptosis, cell proliferation, transcription, and cell migration and in Insulin-like growth factor 1 receptor (*IGFR*), meaning that it can induce hypertrophy of skeletal muscle, collagen formation, collagen biosynthesis, and apoptosis.

It is noteworthy that miRNA-mRNA interaction in rheumatic diseases is scarce, particularly in OA. To date, 20 mRNA have been described that play an important role in OA and the miRNA are known that regulate these [45]; however, functional assays must be approached to verify which of the mRNA implicated in OA are regulated by the miRNA that we describe in the analysis *in silico*. Notwithstanding these results, we open a wide panorama for beginning to approach these.

In conclusion, after profiling 384 miRNA, we were able to detect 12 miRNA in the plasma of patients with primary OA of the knee, and several could regulate or be involved in genes and pathways related with the development of the disease. On the other hand, at least eight of these circulating miRNA are described, to our knowledge for first time, in OA; we think that these miRNA could play a key role in the development and progress of OA, and we also think that these miRNA could be used as biomarkers; however, we are conscious of that one limitation at this time in exploring their uses as biomarkers is the sample size studied.

## Supporting Information

Figure S1 Predicted targets of MicroRNA (miRNA) that are up-regulated in plasma samples of Osteoarthritis (OA) according to an *in silico* analysis by means of the miRecords database.
(TIF)

Table S1 MicroRNA (miRNA) selected for TaqMan profiling Low Density Arrays (TLDA) with highest differential expression between normal patients and patients with Osteoarthritis (OA). About 107 genes were regulated by miRNA.
(DOCX)
