# Supplementary figures and images for: Altered Expression of Circulating MicroRNA in Plasma of Patients with Primary Osteoarthritis and In Silico Analysis of Their Pathways
Source: PLoS One. 2014 Jun 5;9(6):e97690. doi: 10.1371/journal.pone.0097690 (PMC4046959; doi:10.1371/journal.pone.0097690)

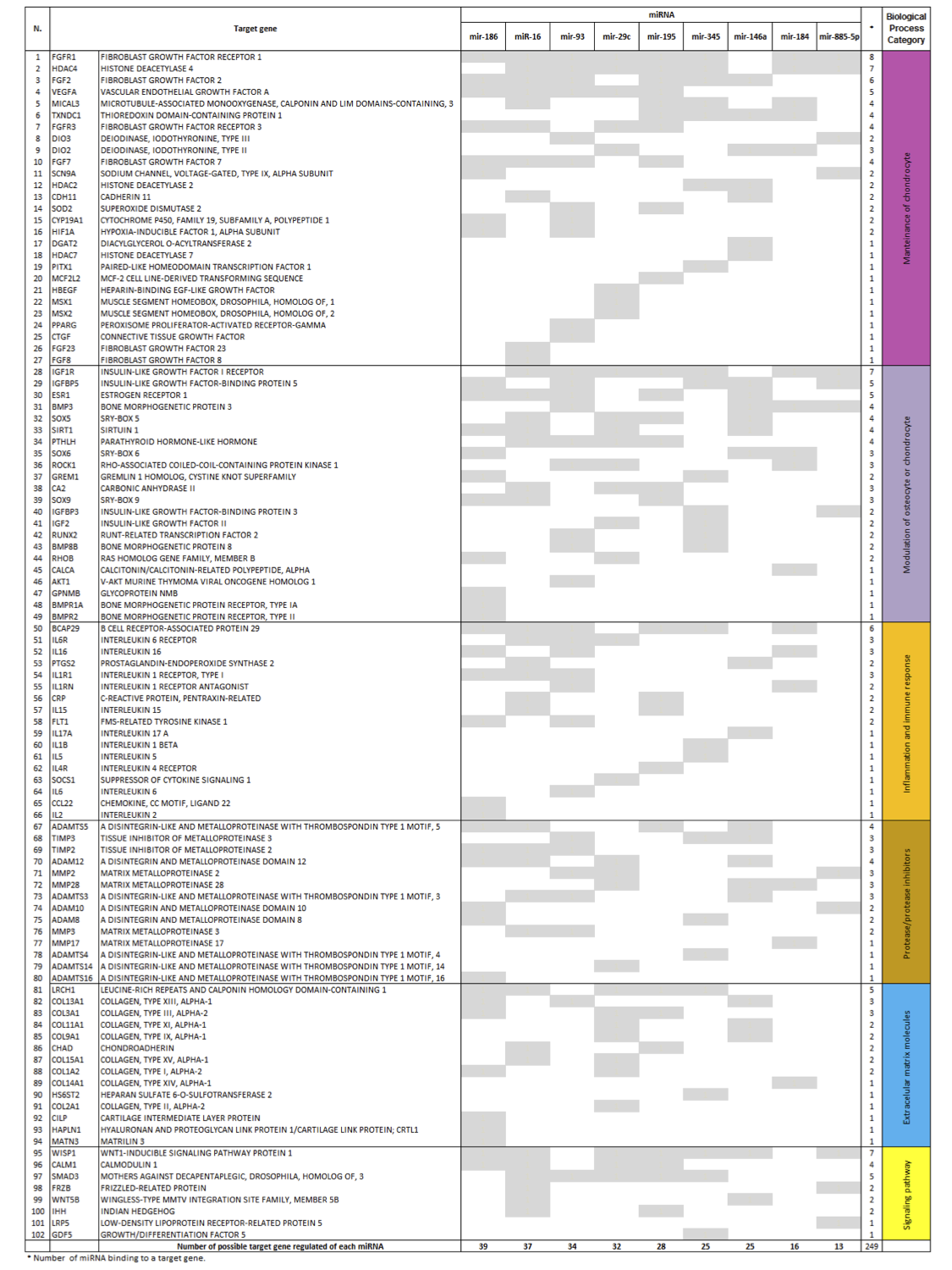

Supplement: Figure S1 — Predicted targets of MicroRNA (miRNA) that are up-regulated in plasma samples of Osteoarthritis (OA) according to an in silico analysis by means of the miRecords database. (TIF) [file pone.0097690.s001.tif]
