# Supplementary material for: Altered Expression of Circulating MicroRNA in Plasma of Patients with Primary Osteoarthritis and In Silico Analysis of Their Pathways
Source: PLoS One. 2014 Jun 5;9(6):e97690. doi: 10.1371/journal.pone.0097690 (PMC4046959; doi:10.1371/journal.pone.0097690)
Supplement: Table S1 — MicroRNA (miRNA) selected for TaqMan profiling Low Density Arrays (TLDA) with highest differential expression between normal patients and patients with Osteoarthritis (OA). About 107 genes were regulated by miRNA. (DOCX) [file pone.0097690.s002.docx]

| **Number** | **miRNA** | ***P* value** | **Log 2-fold change** |
| --- | --- | --- | --- |
| **1** | **hsa-miR-16** | **0.009** | **5.936** |
| 2 | hsa-miR-17 | 0.256 | 1.990 |
| 3 | hsa-miR-19b | 0.213 | 2.637 |
| 4 | hsa-miR-20a | 0.185 | 2.922 |
| **5** | **hsa-miR-20b** | **0.012** | **6.585** |
| 6 | hsa-miR-24 | 0.064 | 3.367 |
| 7 | hsa-miR-25 | 0.706 | 0.731 |
| 8 | hsa-miR-29a | 0.237 | –7.815 |
| **9** | **hsa-miR-29c** | **0.026** | **6.034** |
| **10** | **hsa-miR-30b** | **0.046** | **4.437** |
| 11 | hsa-miR-30c | 0.307 | 1.702 |
| 12 | hsa-miR-92a | 0.692 | 0.642 |
| **13** | **hsa-miR-93** | **0.046** | **4.808** |
| 14 | hsa-miR-106a | 0.148 | 2.247 |
| 15 | hsa-miR-106b | 0.632 | 0.875 |
| **16** | **hsa-miR-126** | **0.048** | **4.031** |
| 17 | hsa-miR-133a | 0.883 | 0.230 |
| 18 | hsa-miR-142-3p | 0.379 | 1.652 |
| **19** | **hsa-miR-146a** | **0.050** | **3.038** |
| 20 | hsa-miR-146b-5p | 0.163 | 3.512 |
| 21 | hsa-miR-150 | 0.154 | 2.818 |
| **22** | **hsa-miR-184** | **0.035** | **2.751** |
| **23** | **hsa-miR-186** | **0.005** | **5.807** |
| 24 | hsa-miR-191 | 0.060 | 4.303 |
| 25 | hsa-miR-193a-5p | 0.620 | –0.829 |
| 26 | hsa-miR-194 | 0.649 | 1.146 |
| **27** | **hsa-miR-195** | **0.032** | **4.338** |
| 28 | hsa-miR-197 | 0.135 | 4.018 |
| 29 | hsa-miR-222 | 0.063 | 5.883 |
| 30 | hsa-miR-223 | 0.111 | 3.007 |
| 31 | hsa-miR-320 | 0.899 | 0.214 |
| 32 | hsa-miR-324-3p | 0.771 | 0.568 |
| 33 | hsa-miR-342-3p | 0.141 | 3.577 |
| **34** | **hsa-miR-345** | **0.030** | **4.315** |
| 35 | hsa-miR-451 | 0.386 | 1.950 |
| 36 | hsa-miR-483-5p | 0.543 | –0.699 |
| 37 | hsa-miR-484 | 0.669 | 0.707 |
| 38 | hsa-miR-486-5p | 0.580 | 0.901 |
| 39 | hsa-miR-518 | 0.085 | 9.294 |
| 40 | hsa-miR-548a-3p | 0.470 | 2.152 |
| **41** | **hsa-miR-885-5p** | **0.050** | **3.229** |
